# Supplementary material for: Human genome diversity data reveal that L564P is the predominant TPC2 variant and a prerequisite for the blond hair associated M484L gain-of-function effect
Source: PLoS Genet. 2021 Jan 19;17(1):e1009236. doi: 10.1371/journal.pgen.1009236 (PMC7845996; doi:10.1371/journal.pgen.1009236)
Supplement: Attachment — Submitted filename: renamed_2bfc6.pdf [file pgen.1009236.s011.pdf]

Point-by-point response to the reviewers' questions:

Reviewer #1: In this manuscript, Böck et al. analyzed the available human genetic sequences and characterized several polymorphisms in the two-pore channel TPCN2 gene. The authors have previously reported that one such SNP (p.M484L) associated with hair color is a gain-of-function variation, with channel harboring the 484L allele generating larger ionic currents than the one harboring 484M. In this paper, the authors report that the channel current enhancement mediated by p.M484L is influenced by another locus at residue 564. TPC2 carrying the major allele at this locus (564P), as previously reported, can be potentiated by the p.M484L variation, but TPC2 carrying the minor allele at this locus (564L) can't. The authors further analyzed the GWAS data and discovered potential linkage of several diseases to variations in this gene.

The data is interesting that the p.M484L gain-of-function variation requires the presence of the major allele at position 564; the analysis is of potential use to link TPC2 variations to some of the diseases. The results however appear to be preliminary and need to be more convincing.

A: We appreciate the reviewer's comments and his opinion that "the data is interesting".

(1) The p.M484L variation has been previously linked to hair color. The authors conclude in this paper that in the 564L background, the p.M484L variation has little effect on the TPCN2 function, suggesting that the p.M484L in the 564L background will not be linked to hair color. The authors should examine the data and see whether such a link indeed does not exists in the 564L background.

A: We apologize if this has not become clear enough in the MS. According to our genomic analysis the combination M484L+P564L does NOT exist in humans. All samples with homozygous M484L variation in TPC2 are on the 564P background. This was one of the surprising findings that variations (heterozygous or homozygous) are almost exclusively found on the 564P background (see Fig.4C-H). In other words, individuals homozygous for 564L barely have variations and only one variation, S681L occurred in a homozygous manner on the 564L background (Fig.4H).

(2) There is generally a lack of mechanism explaining the apparent influence of residue 564 on the potentiation of channel current by p.M484L. Is it because of a change in channel expression level, or in the sensitivity in the activation by PI(3,5)P2 or the inhibition by ATP?

A: The L564P+M484L variant shows a higher sensitivity to PI(3,5)P_2 than the P564L+M484L variant as shown in Fig.5A and B. The L564P variant alone shows no change in PI(3,5)P_2 sensitivity compared to P564L. Current densities are comparable. Expression levels and subcellular localization of all variants have now been analysed by WB experimentation and confocal microscopy (see new Fig. S6) to rule out that expression plays a role. None of the SNPs was found to be mislocalized. They are all expressed in lysotracker positive vesicles according to confocal image analysis with no significant differences in colocalization (PCC, Mander's). We also found no significant differences in expression using WB, rather GOF mutants appeared to show a trend towards lower expression. This is in line with previously published results (Chao et al., PNAS, 2017 Suppl. Fig. 1C and D). Importantly, no significant differences were found for M484L+L564P versus M484L+P564L. It can thus be ruled out that expression or mislocalization are the underlying causes for the observed GOF effects or the differences between M484L+L564P and M484L+P564L.

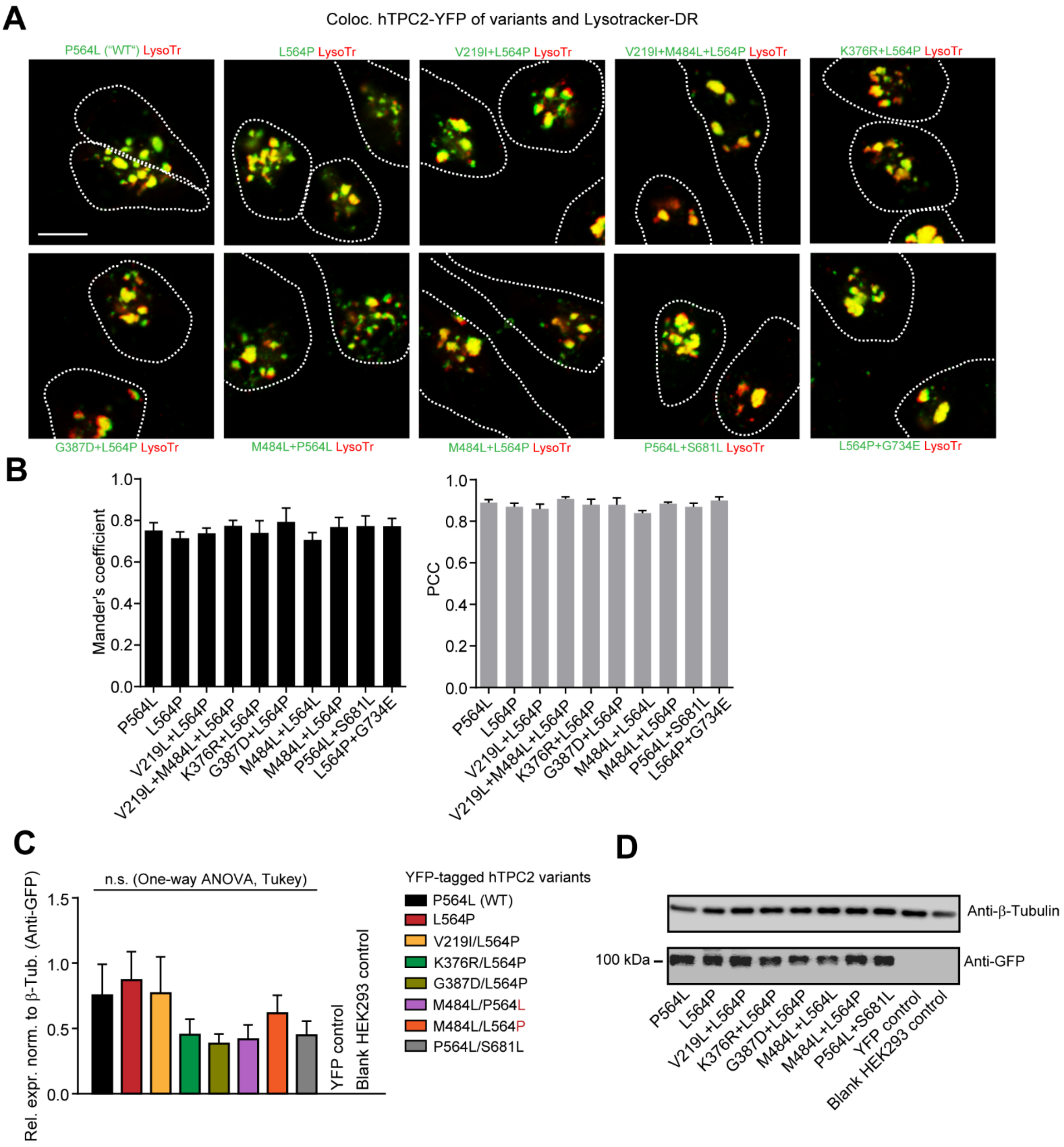

To gain further mechanistic insight, we have introduced alternative mutations at position 564 (L564G and L564A) to assess if the hypothesis that helix-breaking amino acids are leading to the observed effect while a non-helix breaker such as A has a similar effect as L. As a result we found that only a strong helix breaker such as proline at position 564 is capable of mediating the GOF effect (see new Fig.5H-J).

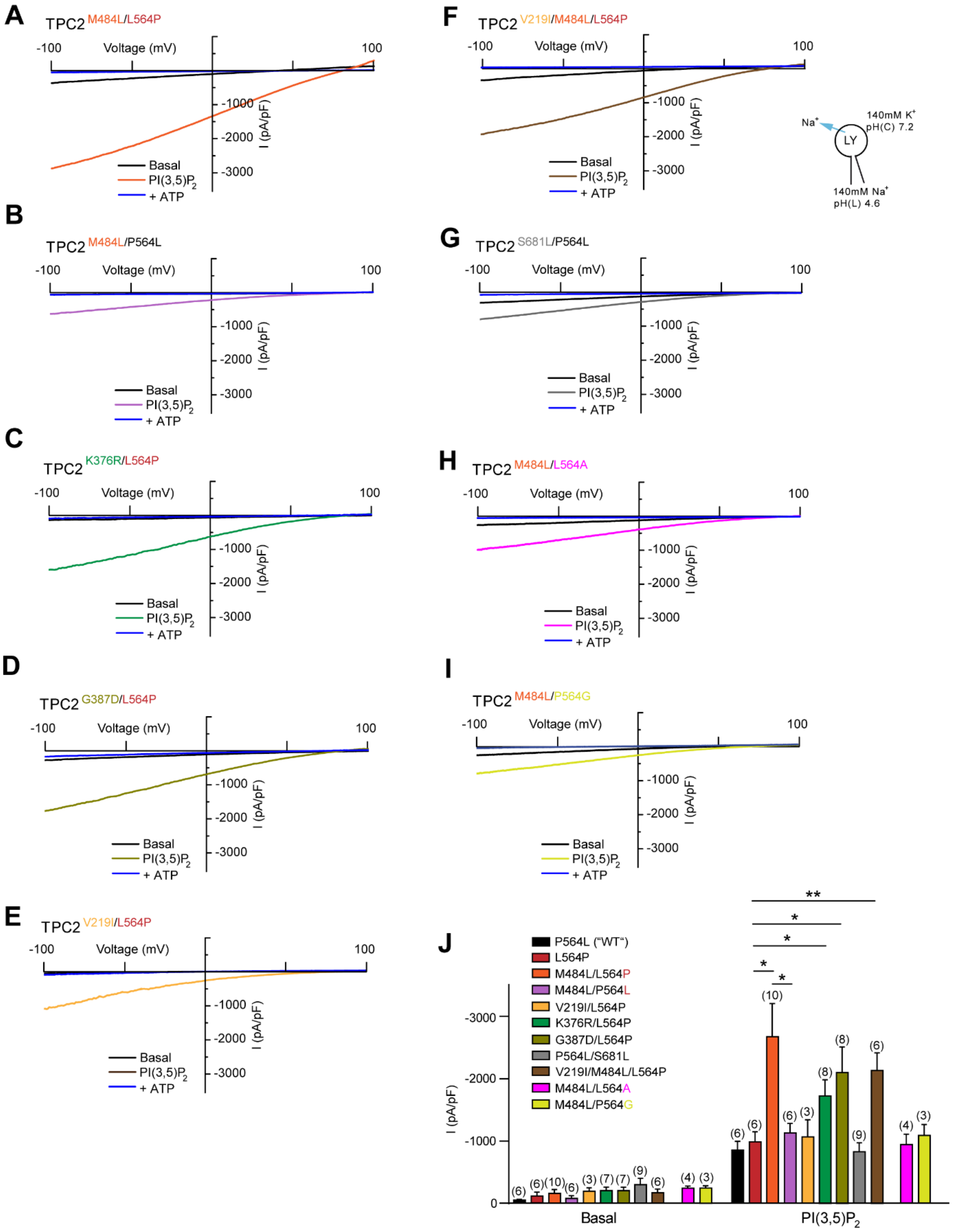

We further mutated amino acids up- and downstream of 564 and introduced prolines (K563P, M565P) to assess the possibility that prolines up- and downstream of 564 may have similar effects as proline at position 564. This however was not the case (see new Fig. S7). In sum, the data suggest that the GOF effect does require both variations to occur simultaneously at these two particular positions in the TPC2 protein. In their hTPC2 cryo-EM structure paper of 2019 She et al. (Elife, 2019) showed in Fig.5B that structural changes in the IIS4-S5 linker region containing 564P occur when switching from closed

to open state and vice versa. Based on the structure, it is evident that the L564P site is situated directly between M484L and the channel pore. We therefore assume the M484L-associated GOF to be transduced through the L564P-encoding IIS4-S5 linker, dilating the channel pore further upon PI(3,5)P2 activation. The IIS4-S5 linker appears necessary to provide space for pore dilation and channel opening. Substituting the helix-initiating P564 with a leucine would dramatically affect this linker helix extension. We propose a model where M484L is amplifying the effect of PI(3,5)P2 activation, requiring signal transduction through L564P to result in pore dilation. A more elaborate investigation of the interplay between M484L and L564P would be however outside the scope of this paper. While being intriguing, the methods required (comparative cryo-EM of polymorphic channels and/or molecular dynamics simulations) would be both time-consuming and resource-demanding, and outweigh the main focus of this paper, i.e. a complete electrophysiological characterization of a tremendously polymorphic endolysosomal ion channel.

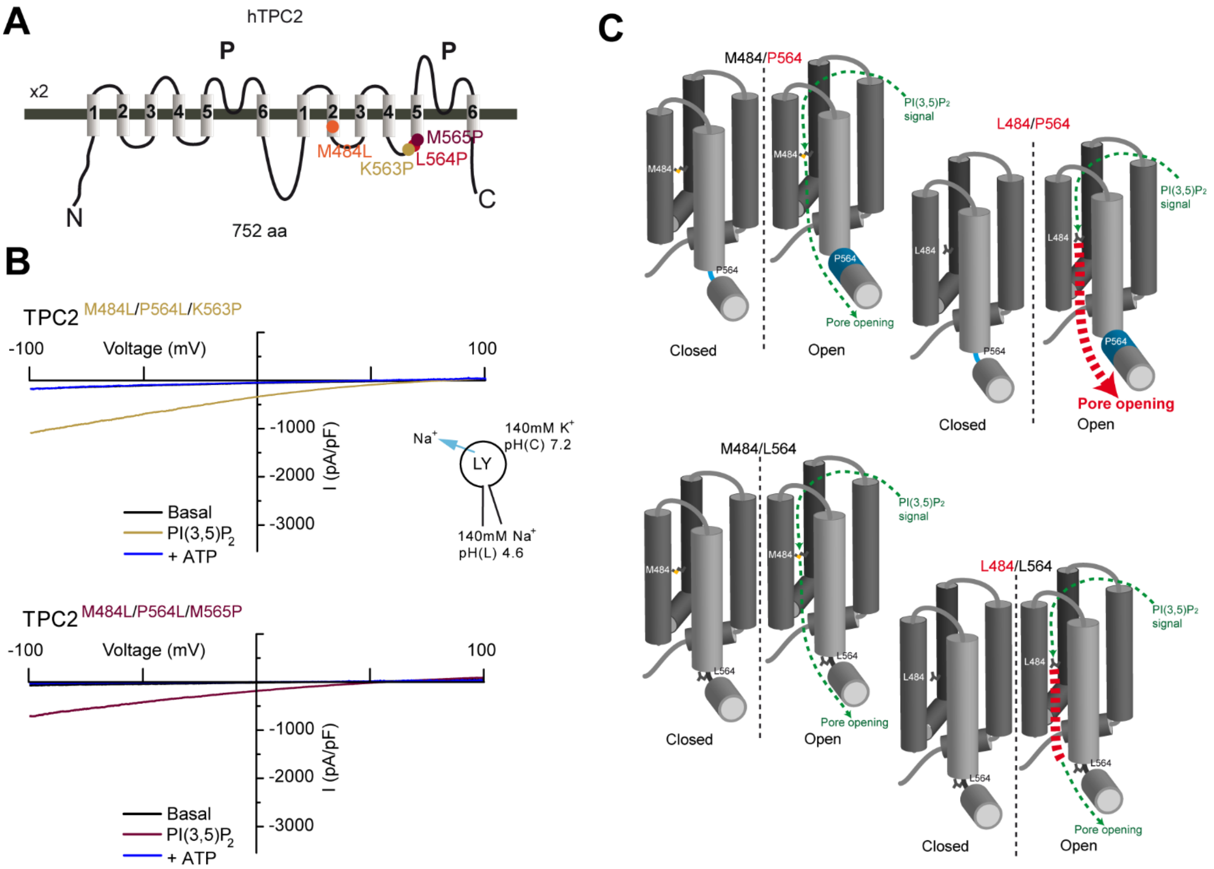

(3) Most of the functional analysis is from overexpression. There is limited data from the analysis of SNP-associated endogenous TPC function from cultured fibroblasts, and it is not that convincing due to the limited number of recordings (n=3-6). In addition, the 9 donors analyzed in Fig. S6 all harbor 564P and therefore can't be used to test the effect of 564L. In order to test the effect of 564L on endogenous TPC2 function, the authors should analyze more fibroblasts and identify individuals carrying the minor allele. Given the fairly high frequency of the minor allele (~13%), this might be doable. Alternatively, even better, the authors can perhaps knock-in the 564L minor allele into one of the existing fibroblast cell line and analyze the effect.

A: TPC2 currents are generally small in fibroblasts. Nevertheless, we were able to convincingly recapitulate key overexpression results in the donor fibroblasts, in particular the discovery of novel GOF variants, K376R and G387D was confirmed (please see new Fig. S8). We have also increased the n numbers for these fibroblast lines now. Regarding human donor fibroblasts with P564L+M484L, we would like to point out again that this combination does NOT exist in humans (see Point 1). Hence, human fibroblasts with the combination P564L+M484L are unfortunately not available in any population. Regarding human donors carrying only P564L, it is clear from our analysis (please see Fig.1 world map) that this variant barely occurs in European samples. We have screened a total of 136 (!) donor samples. None of these carried the P564L variation in a homozygous manner. Screening more individuals would require again approval by the ethics committee of the LMU. Considering the time for recruitment of donors this would take several months. In addition, recruitment of indigenous people from South America or South East Asia would much more likely result in the right genotype but access to such samples seems not feasible in a reasonable time window. Likewise, a CRISPR/Cas9 approach would require several months of work from generation of the cell line to analysis by endolysosomal patch-clamp. From our perspective, the patch-clamp data in HEK293 cells have given a very clear result regarding M484L+P564L versus M484L+L564P currents and given that other data were reproducible 1:1 in the fibroblast lines, we have no reason to doubt these results.

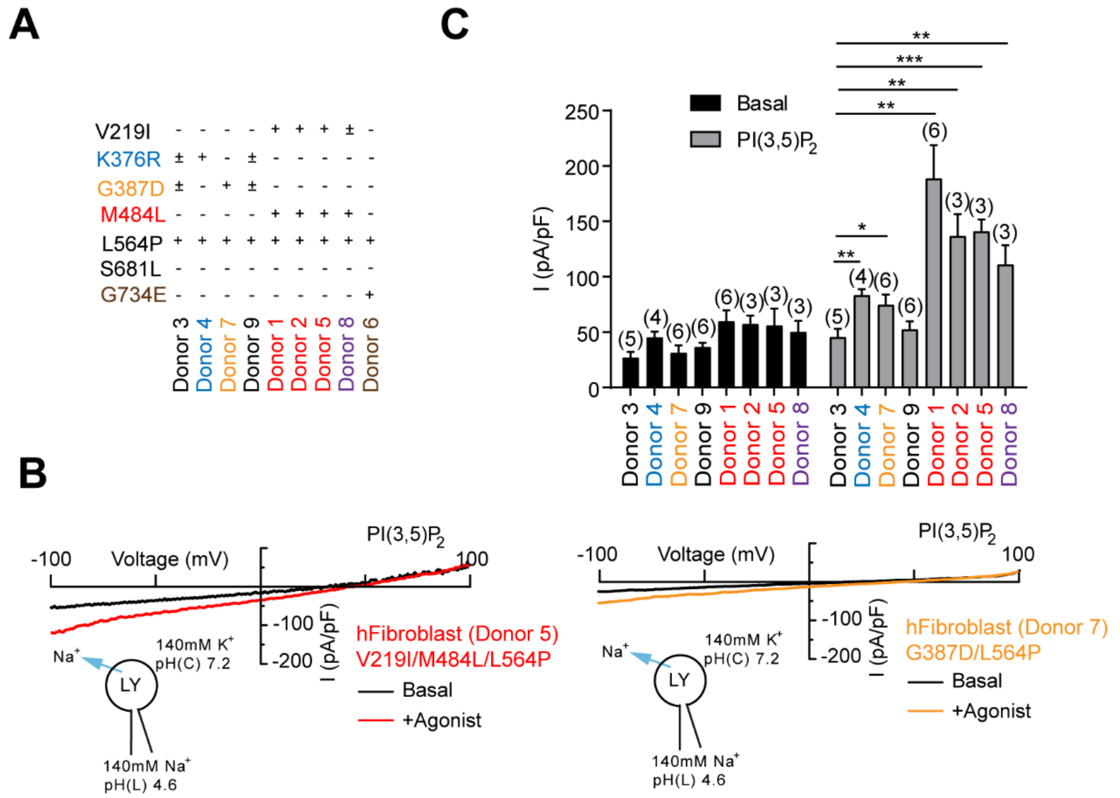

Reviewer #2: This study explores the genetic and functional analysis of polymorphisms found in the endo-lysosomal cation channel TPC2. They used publically available dataset of human genome sequences to determine the polymorphism profiles and frequencies across the populations represented in the datasets. They then used endo-lysosomal patch-clamp analyses to show that the GOF M484L Variant only acts as a GOF variant on the 564P but not on the 564L background. The also compare activity in donor fibroblasts samples and find suggest that there are TPC2 variation patterns across the globe that alter TPC2 to become more or less active with and/or without stimulation. This is an important characterization of a gene with significant medical and global importance.

A: We thank the reviewer and appreciate his view that "this is an important characterization of a gene with significant medical and global importance."

Comments:
Page 9. The authors state that there is a higher variation frequency for TPC2 than for similar channels. While it is true that there more polymorphisms found for TPC2 than for other channels I am not sure if this is significant. Did they control for exon size and look at noncoding/intronic region variation of these genes to make this statement. If not maybe just state the frequencies without a qualifier as it being higher variation. Maybe say the most variation was seen in.

A: We have now calculated the percentage of polymorphisms (see Fig. F below; left: all SNPs; right: only homozygous SNPs) for TRPML1, 2, 3 and TPC1, 2, relative to the coding sequence length for each gene. We omitted intron lengths from this analysis as intron variations were not a focus of this study.

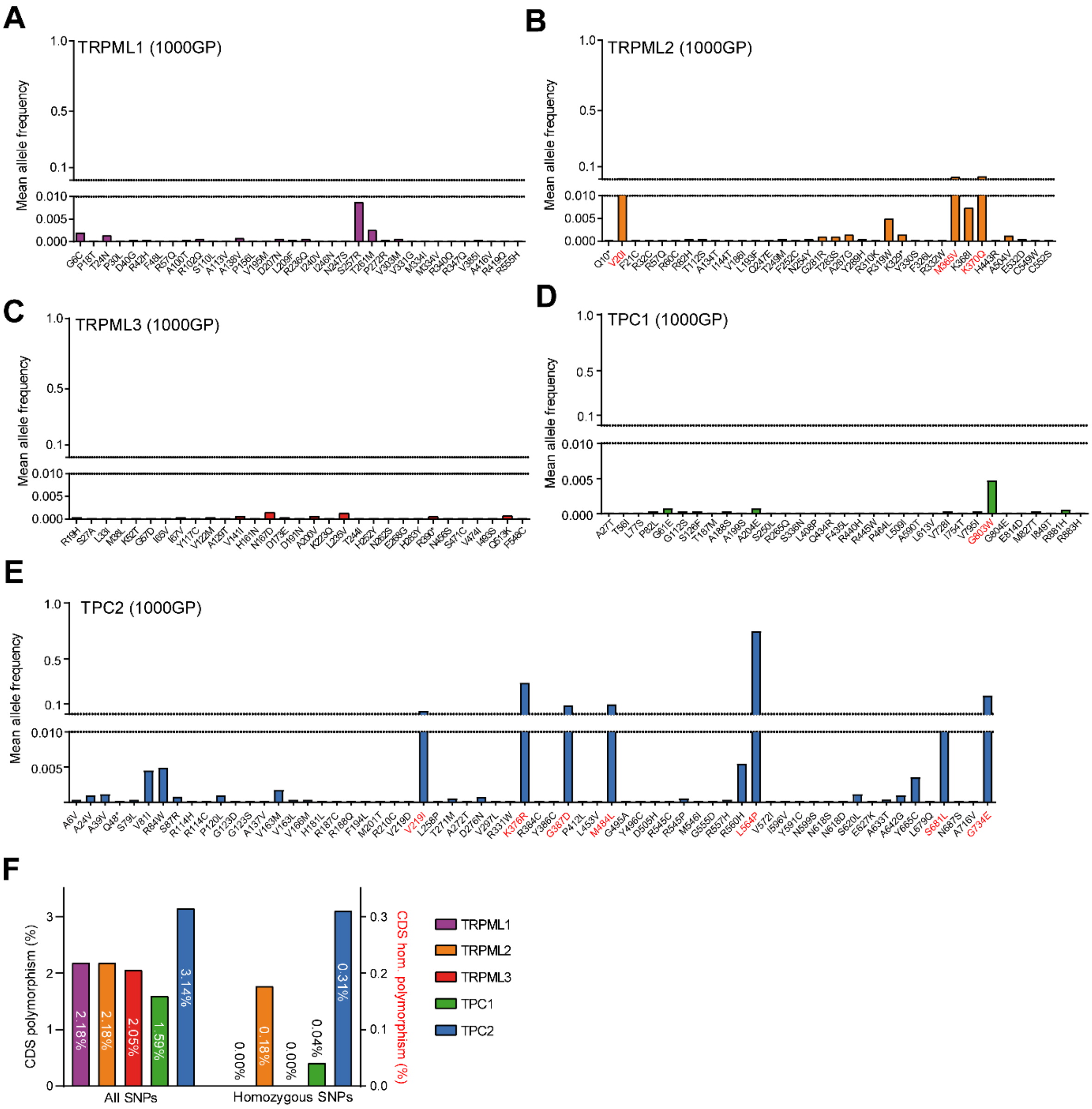

In transfection and endogenous patch clamp studies, how did authors control for equal protein levels and correction cellular locations of TCP2 variant?

A: By measuring current densities = current in pA divided by capacitance in pF we can normalize to vesicle size and thus control indirectly for the average number of channels measured. All channels are localized to lysosomes, otherwise it would not have been possible to patch-clamp them (if e.g. mislocalized to ER or cytosol). We have also confirmed this now by confocal image analysis (see new Fig. S6). In addition, we have now performed a WB analysis to assess expression levels (see new Fig. S6). We found no significant differences in expression, rather GOF mutants appeared to show a trend towards lower expression. This is in line with previously published results (Chao et al., PNAS, 2017 Suppl. Fig. 1C and D). Importantly, no significant differences were found for M484L+L564P versus M484L+P564L. It can thus be ruled out that expression or mislocalization are the underlying causes for the observed GOF effects or the differences between M484L+L564P and M484L+P564L.

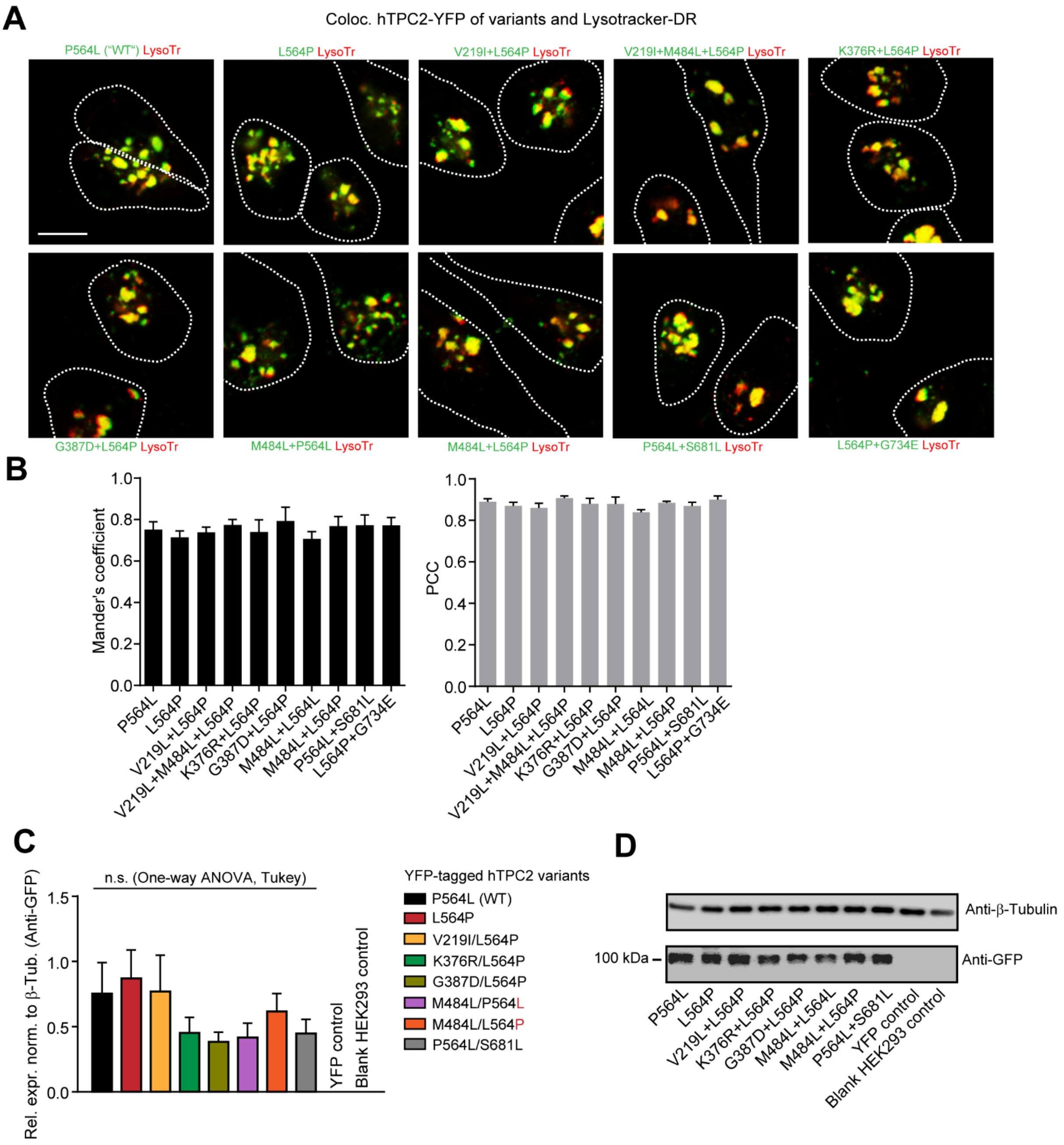

PAGE 11. The authors stat that several fibroblasts were assessed. Please insert the actual number.

A: We have replaced "several" with the actual number "nine" in the text.
